# Development and validation of the ND10 to measure neck-related functional disability

**DOI:** 10.1186/s12891-022-05556-7

**Published:** 2022-06-23

**Authors:** Joy C. MacDermid, David M. Walton

**Affiliations:** 1 Roth | McFarlane Hand and Upper Limb Centre, St. Joseph’s Health Care, 268 Grosvenor Street, London, ON N6A 4V2 Canada; 2 School of Physical Therapy, Western University, London, ON Canada

**Keywords:** Neck, Cervical, Disability, Activity, Development, Evaluation, Health-related quality of life

## Abstract

**Background:**

Previous neck-specific patient-reported outcome measures (PROMs) have tended to measure both symptoms and disability. This multi-staged study developed and evaluated a neck-specific PROM focusing on functional disability.

**Methods:**

This study integrated findings from systematic reviews on neck-specific outcome measures, patient interviews, qualitative studies on neck disability, and iterative item testing to develop a 10-item measure of neck-related disability (ND10). Content validity was assessed by classifying items using the International Classification of Functioning, Disability and Health (ICF) and perspective linking. Patients (*n* = 78) with neck pain completed cognitive interviews, exploring items of the Neck Disability Index (NDI) and ND10, and completed structured questions related to literacy and relevance. Test–retest reliability and internal consistency were evaluated using intraclass correlation coefficients, Bland Altman graphs, and Cronbach’s alpha. Concurrent convergent validity was evaluated by comparing the ND10 to the NDI, Single Assessment Numeric Evaluation (SANE), and Disabilities of the Arm, Shoulder and Hand (DASH). Known group validity was determined by comparing ND10 scores from patients, who rated their neck as more or less than 1/2 of “normal” on the SANE, using t-tests.

**Results:**

The ND10 requires respondents to make rational judgements about their neck-related body function and disability. It has high internal consistency (0.94) and re-test reliability (0.87; SEM = 3.2/100; MDC = 7.5); and no re-test bias (mean re-test difference of 0.6). It followed expected correlation patterns, being highly correlated with related multi-item PROMs (*r* = 0.85–0.91), and moderately correlated to the single-item SANE. More patients agreed that the ND10 was easily readable than did so for the NDI (84% vs 68%; *p* < 0.05). All the PROMs distinguished the patients who perceived themselves as being abnormal/normal defined by a dichotomized SANE (*p* < 0.01).

**Conclusion:**

The ND10 is reliable and valid for measuring neck-related functional disability. Longitudinal and cross-cultural translation studies are needed to support future use.

**Supplementary Information:**

The online version contains supplementary material available at 10.1186/s12891-022-05556-7.

## Introduction

Neck pain is one of the most common musculoskeletal disorders with one third of all adults experiencing it during the course of one year, and 70% doing so over the course of their lifetime [1]. The severity of disability can range from minor to severely debilitating and the natural history is characterized by episodic reoccurrence [2, 3]. Radiologic [4] or physiologic measures [5] rarely explain neck pain. As a result, accurate measurement of symptom severity and functional disability is essential to targeting treatment and evaluating treatment outcomes. Systematic reviews indicate that baseline pain and disability are the most potent prognostic indicators of future pain and disability outcomes [6, 7].

Musculoskeletal health outcome measures are commonly used to evaluate symptoms, disability, and quality of life, and how this change following an intervention. Neck disorders can cause pain [8, 9]; and disturbances in joint motion [10, 11], sensory function [12–15], proprioception [16, 17], motor function [18–20], coordination [5, 21], posture [22, 23], and balance [23, 24]. These can lead to functional disability [2, 25, 26], participation restrictions [27], reduced work capacity [28–30], and lower quality of life [31, 32]. There are a variety of impairment and disability measures that have been designed to assess these different constructs [23, 33–38]. A survey of international practice patterns of clinicians with respect to assessing the outcomes for patients with neck pain indicated that the Numeric Pain Rating Scale (NPR) (a single item on pain [37]), the Neck Disability Index (NDI), and the Disabilities of the Arm, Shoulder and Hand (DASH) (developed for upper extremity [39]) are the patient-reported outcome measures (PROMs) most commonly used by clinicians [40].

The two primary features of musculoskeletal conditions, including neck disorders, are pain and disability. Content validity of PROMs requires a clear conceptual foundation with a defined construct [41, 42]. Increasingly, there have been moves to define conceptual frameworks for identification of core constructs as a preliminary step to improving measurement in the field of musculoskeletal disability. A recent international consensus panel [43] identified 6 core domains for whiplash-associated disorders: Physical Functioning, Perceived Recovery, Work and Social Functioning, Psychological Functioning, Quality of Life, and Pain. Many existing measures have not adequately defined a single construct, but rather sample across multiple constructs or domains within a global construct or health condition. A recent international outcome measure core set consensus panel for whiplash disorders concluded that “*the content validity of these PROMs has yet to be established… and until this is undertaken, it is not possible to recommend 1 PROM over the other*” [35]. Commonly, PROMs for musculoskeletal conditions include items on pain and function and compute total scores—as if these items reflect a single construct. Combining symptoms and disability in a single score from PROMs may not be justifiable on psychometric rounds since these may not represent a single construct. Furthermore, combining different scores together may undermine clinical reasoning or research hypothesis testing since being able to differentiate the impact of interventions on specific constructs is critical to problem-solving and hypothesis testing. Where adequate content validity is not present, measures do not provide accurate information about what aspect of patient status is changing over time [44]. Content validity is a prerequisite to other psychometric properties like factor validity and unidimensionality [45]. Finally, consensus panels have verified that pain and disability are separate constructs that are important core outcomes in health conditions causing neck pain [43].

A variety of PROMs have been previously established. The most of commonly used is the NDI developed by Vernon and Mior [46]. It was constructed based on 5 items adapted from the Oswestry Low Back Pain Index (OLBPI) and an additional 5 new items [46]. The developer published a summary paper in 2008 summarizing a 17-year history with the NDI [47], reflecting its position as the earliest and most commonly used neck-specific PROM. Systematic reviews of the measurement properties of the NDI concluded that there was a deep pool of evidence supporting the NDI as being reliable and responsive, but found validity concerns about the factor structure, and relevance given the number of items left missing in certain populations [33, 48]. Although the NDI is used as if it provides interval-level scaling, Rasch analyses indicate that is not achievable with the original measure [49–51]. Further, there are substantial differences between the 2 proposed Rasch-based scoring version and the original [52]. A variety of neck-related PROMs have been developed subsequently. An overview of neck-related PROMs [48] found that more limited research on the other neck-related measures: Northwick Park Neck Pain Questionnaire (20 items), Copenhagen Neck Functional Disability Scale (15 items), Neck Bournemouth Questionnaire (7 items), and Neck Pain and Disability scale (20 items).

Construct clarity is important in outcome evaluation. International consensus has concluded that functional disability is an important construct for assessing outcomes in neck-related health problems [43]. However, the wording of many of the current neck PROM items suggests that they measure neck-related pain interference–how much neck pain interferes with function. Pain interference and disability are related but different constructs. It may be problematic when PROMs conflate pain and function or do not specify what they are measuring is pain interference. This construct ambiguity might explain why some factor analyses studies indicate that the NDI contains 2 factors [53–56]. This is further supported by qualitative studies of experts and patients who suggest that the NDI measures more than physical functioning [44]. Since physical functioning is 1 of the core constructs agreed upon by an international panel [43], a measure that focuses solely on function/disability for people with neck conditions is needed. A recent review of disability measures for whiplash concluded that “the content validity of these PROMs has yet to be established…, *and until this is undertaken, it is not possible to recommend 1 PROM over the other for inclusion in* (core outcome measure sets)” [35].

Although there are several PROMs used for patients with neck pain, there is no measure that limits its focus to functional disability. Some neck-related PROMs measure symptoms, functional disability, pain interference, and/or quality of life [48]. Surveys [40, 57] suggest that the DASH is frequently used to measure the upper extremity-related components of neck pain which are not covered by the NDI, despite the fact that the DASH was not developed for this purpose. The importance of the upper extremity in neck-related functional disability was emphasized by qualitative studies which found that this was an important component of neck symptoms and disability from the patient perspective [27]. Lab-based studies have demonstrated altered upper extremity neuromuscular functioning in people with neck pain [21], which confirms the importance of considering upper extremity functioning in neck conditions.

The lack of sufficient involvement of patients with neck pain in developing some of the early neck-specific PROMs may have contributed to important gaps in the scope of symptoms or disability included on the NDI. Content validity requires that during development and validation the relevance of items be assessed with respect to the target population [41]. When PROMs fail to address important elements or the full scope of a construct, then content validity is inadequate, regardless of whether the measure demonstrates adequate quantitative psychometric properties.

Therefore, the purpose of this paper is to report the development and validation of a PROM that is designed to measure neck-related disability in patients with neck pain/disorders. Specific objectives are to describe the development process, content validity, readability, potential for floor/ceiling effects, test–retest reliability, and construct validity.

## Methods

### Scale conceptual definition

The Neck Disability 10 (ND10) was developed based on analyzing gaps in current neck PROMs using qualitative studies with patients living with neck pain and quantitative studies on neck disability. Guiding principles were developed to avoid problems identified in previous PROMs that measure the construct of neck-related disability:

1. The items should focus on the single construct of neck-related disability.
2. Valid legacy constructs of neck-related disability from prior PROMs could be retained if they were confirmed by patients as being relevant and re-worded for clarity as needed.
3. New salient items from patient-based qualitative or quantitative studies were added to the item pool to address gaps in current PROMs.
4. Health literacy, potential for translation across groups/cultures, and cognitive burden were considered in item bank refinement and decisions on format.

### The ND10

After iterative item selection with patients and experts, mapping legacy, and new items in the item pool, and pilot testing of items, the final version of the ND10 is presented as Supplementary File 1. The ND10 is a 10-item scale that measures neck-related disability. Each item is scored on a scale from zero (no difficulty) to 5 (unable to do at all). The scale is scored calculating a percentage out of 100 (if no missing items, then total score can be multiplied by 2). If items are missing, the total score is calculated as a percentage to range from 0–100 points. The rationale for remediated legacy items and new items from the iterative consultative steps to refine the item bank to the final set of items is summarized in Table 1.

**Table 1:** Content validity of ND10: ICF and perspective linking of content

Structural Decisions	No difficulty	A little difficulty	Moderate difficulty	A lot of difficulty	Extreme difficulty	Unable to do at all
	- A happy face was used to reduce confusion about direction/nature of scale and lower the literacy/cognitive burden- Consistent descriptors lower literacy/cognitive burden- The focus on a single concept (disability) allows for the use of the same Likert metric (amount of difficulty) across all items					
Items	Content Validity Rationale				ICF Codes	IPC
Do my personal care (washing, dressing etc.)	- A legacy construct from NDI (item #2), and DASH (items #13 and 14) - considered a low difficulty item [58] - relevancy established in patient interviews [27]				D5- self care	RBp
Lift and carry heavy objects	- NDI legacy construct (item #3) “lifting” and DASH #11 - “and carry” added based on qualitative interviews [27] and data from DASH items [58]				d430 lifting and carrying objects	RBc
Read (a book, paper, or electronic device)	- NDI legacy construct (item 9-reading) - Patients noted that usual “Reading” often meant from electronic device				d4401 Grasping- d166 Reading	RPm
Do my usual work (or role)	- NDI legacy item #4, DASH #23 - Difficulty at work a common patient concern [27, 59, 60]				d840-d859 Work and employment	RSc
Drives in a vehicle (car, bus, train etc.)	- NDI legacy construct (item #8)—“driving” has a high rate of missingness; and DASH #20 (transportation) - the ND10 item includes different vehicles or being a passenger in a vehicle ND10 tested “long drives” to better define exposure, but less clar to patients				d470 Using transportation; or driving (d475)	RIp
Do my usual recreation	- NDI legacy construct (item 10) and DASH items #17 and #18 - ND10 clarifies as usual recreation - endorsed by participants [27, 59, 60] and DASH results [58]				d920 Recreation and leisure	RSc
Concentrate on tasks	NDI item #6 addresses concentration; endorsed in patient interviews [27, 59, 60]; focus “on tasks” added to link more clearly to function				b140 Attention functions	RPp
Sleep	- NDI legacy construct (item #7 -time sleep disturbed) and DASH #29 - ND10 tested difficulty sleeping in usual position rather to distinguish from sleep quality – but this was more confusing/double barrelled so return to simple version				b134 Sleep functions; Changing and maintaining body position (d410-d429)	RBp
Place an object on a high shelf	- DASH legacy construct (item #6) - moderate difficulty in patients with neck pain [58]				d4452 Reaching	RBc
Do overhead work—(like change light bulbs, painting/washing walls)	- DASH legacy item #12 was rated as difficult by patients [58]				d445 Hand and arm use; d415 Maintaining a body position	RBc
ICF Response Options	- categorization of the response options in ICF				All items on one metric of intensity (amount of difficulty)	

Legacy constructs do not have the same wording on the NDI and DASH. NDI legacy items tended to link pain and function in a single question. ND10 does not address pain

### Comparison study measures

#### The Neck Disability Index (NDI)

The NDI is a 10-item PROM that assesses neck-related pain interference with function [33, 46, 48]. It was expected to be concordant with the ND10 based on specificity to neck disorders. Two Rasch-based versions of the NDI exist and show systematic differences from the traditional ordinal NDI [52]. The NDI-5 is a Rasch-based, 5-item version of the NDI [50] developed to focus on the subset of NDI items that address function and provide interval-level scaling and was selected as most comparable in the intended construct: neck-related disability. NDI-5 scores can be represented two ways: as a raw score and using the Rasch-based transformation that provides interval-level scaling.

#### The Quick Disabilities of the Arm, Shoulder and Hand (QDASH)

The QDASH is an 11-item measure of upper extremity symptoms and disability. It was selected as a comparator as it has been shown to be salient to people with neck disorders [58], since patients report that neck pain and/or upper extremity movement affects their neck pain [27]. Since upper extremity items were one of the gap areas identified in qualitative research [27], it was seen as important to consider this construct.

#### The Single Assessment Numeric Evaluation (SANE) for neck

The SANE is a single global item [61], first reported for use to evaluate function in patients with knee problems, and subsequently applied to a variety of health conditions and body areas [36, 62]. The patient responds to “how would you rate your (body area) today as a percentage of normal (0% to 100% scale with 100% being normal)”. It has been validated for multiple musculoskeletal conditions [61, 63–67]. Based on previous studies we expected a moderate relationship between the ND10 and the SANE [62, 63].

### Patient recruitment

Patients with neck pain were recruited through physiotherapy clinics. Exclusion criteria included lack of ability to complete questionnaires in English. The study was approved by the Hamilton Integrated Research Ethics Board and all respondents provided informed consent.

#### Data collection

Respondents completed the full version of the ND10, DASH, and the NDI on a single occasion. For the test–retest data, the respondents were asked to complete the ND10 for a second test occasion and return the survey within 14 days. The SANE was also completed on the second test occasion. The NDI-5 and NDI-5 T were extracted from the full NDI, and the Rasch scoring applied [50]. The datasets used and/or analyzed during the current study are available from the corresponding author on reasonable request. A talk-aloud approach cognitive interview with follow-up probes approach was used to explore respondents’ perceptions of individual items [68] in 15 patients.

### Analyses

#### Content validity

Content validity was integrated in the development process and informed revisions of the items. New items were derived from the published qualitative and quantitative literature on the experience of neck disability, including a specific qualitative study designed to assess the experience of neck pain and its contributors [27]. Iterative feedback was obtained from people living with neck pain and measurement experts to revise the items to ensure clarity. Structured content analysis of the final version of the ND10 was performed using 3 methods. The content of the ND10 was compared to other neck-related PROMs that have been reported in the literature. Secondly, the International Classification of Functioning Disability and Health (ICF) linking procedures were used to code item content according to established linking rules [41, 69, 70] to specific ICF codes. ICF linking provides a mechanism to communicate content in a common international language and is particularly salient to measures of disability. Item linking was performed by 2 raters, using updated rules that include perspective and response options. Item Perspective Classification (IPC) was used to classify the nature of the decisions made in responding to individual items [71]. A two-level IPC was used which focuses on whether it was a rational or emotional judgement; and if the question addressed psychological, social, biological, or inorganic issues/content.

#### Scale distributions and floor/ceiling

Box plots were used to examine the distribution of scores for individual items and subscales. We adopted the commonly used 15% threshold for patients achieving the highest and lowest score to define a ceiling and floor effect (i.e., scores of 0–10 and 90–100).

#### Reliability and Agreement

The following statistics were calculated:

- Internal consistency
- Reliability: intraclass correlation coefficients (ICC) (2,1) [72]
- Agreement: Bland and Altman graphs to determine potential bias (mean difference across test occasions) and limits of agreement [73, 74]
- Standard error of measurement and minimal detectable change (90% confidence)

### Construct validity

The following hypotheses were constructed to assess construct validity. The expected relationships were then assessed using Pearson correlations.

1. The ND10 should demonstrate high correlation (i.e., convergent validity indicated by *r* > 0.75) with the NDI, NDI-5, and DASH, given conceptual concordance and prior research demonstrating correlations between the NDI and DASH.
2. The SANE would correlate moderately with the ND10, given that it is the single item rating of “normality” and expected to be less directly related to the construct in a multi-item neck disability measure.
3. The people who see themselves as less than 50% “normal” on the SANE will have higher ND10 scores (discriminative, known-groups validity).

### Patients’ preferences

Patients’ preferences were addressed by questions completed immediately after completing the NDI and ND10 (random order). Patients were questioned about the clarity and relevance of the two questionnaires.

## Results

The ND10 was developed as a 10-item functional scale for patients with disorders of the neck. The final version is presented with scoring instructions as Supplementary File 1. Characteristics of participants in the validation studies are listed in Table 2 and indicate a 75/25 female imbalance in gender distribution.

**Table 2:** Participant characteristics

Measure	Mean	SD	n
Gender	75% women; 25% men		
Patient-reported source of neck pain	Trauma 60%		
	Disk Problem 19%		
	Pinched Nerve 15%		
	Arthritis 17%		
	Muscle Strain 40%		
	Don’t Know 13%		
Age (years)	47.5	13.8	78
Length of symptoms prior to evaluation (years)	6.5 (Median 3.0)	8.7	78
ND10 score (/50)—Time 1	30.6	20.5	78
ND10 score (/50)—Time 2	26.2	20.5	36
NDI (/50)	31.8	17.9	78
QDASH (/100)	35.5	22.3	51
NDI-5 (Rasch transformed)	21.7	16.5	30
SANE (/100)	65.4	22.6	72

### Content validity

Item content comparison of neck-related PROMs indicate some common functional items across six neck-related PROMs (e.g., personal care, driving, lifting, sleep, and work) ask how neck pain influences function rather than purely rating functional difficulty/ability (Table 3). Some of the PROMs have more emphasis on physical symptoms like motion or paresthesia, or mental symptoms like anxiety or depression. Other PROMs include other constructs like social functioning, medication use, or attitudes about the future.

**Table 3:** Comparison of item content of ND10 to other neck-specific PROMs

ND10 Disability Items	NDI(10)	NPAD(20)	NPNPQ(10)	CNFDS(15)	NBQ(7)
Washed and dressed	√P	√P		√ √P	
Lift and carry heavy things	√P		√P	√P	
Read (book, paper, tablet, computer, or phone)	√P		√P	√P	
Do my usual work	√	√P	√P		√P
Drive or ride (car, bus, train, bicycle etc.)	√P	√P	√P		√P
Do my usual recreation or sports	√P	√P			
Concentrate on tasks	√	√P		√P	
Sleep	√	√P	√P	√P	
Place something on a high shelf					
Do overhead work (Like change light bulbs, wash walls)					
**Disability Items from other PROMs not on ND10**		√P			
Daily activities				√P √H	√P
Standing		√P			
Walking		√P			
**Out-of-construct items from other PROMs not on ND10**					
Pain	√	√√√	√		√
Headaches	√			√P	
Social life		√P	√P	√P	√
Emotions		√P			
Anxiety					√
Depression					√
Control of pain					√
Stiffness		√P			
Neck motion		√P VP			
Pins and needles			√		
Duration of symptoms			√		
Global rating of change			√		
Time at home				√P	
Time in bed				√P	
Emotional relationship with family		√P		√P	
Effect on future		√P		√P	
Medication use		√P			

This table shows the extent to which the same or similar items are contained across different neck disability PROMs (√). A “P” added to the “√” indicates the item mentioned a functional task, but was framed in terms of neck pain interference with function (not functional (dis)ability). An “H” indicates framed in terms of help needed

*ND10* Neck Disability 10, *NDI* Neck Disability Index, *NPNPQ* Northwick Park Neck Pain Questionnaire, *CNFDS*, Copenhagen Neck Functional Disability Scale, *NBQ* Neck Bournemouth Questionnaire, *NPAD* Neck Pain and Disability scale. The number of items is in brackets after the PROM acronym

ICF/IPF codes and item content validity coding for the ND10 are presented in Table 1. IPF codes indicated that 100% of the items involved rational decision; 5 (half) of the items focused on the biological domain; 3 psychological; 1 social; and 1 on inorganic content. The ICF linking revealed that all ND10 items were linked to unique ICF codes: 2 of the items linked to body functions (sleeping, concentration), while the remaining 8 items were linked to disability codes. Disability items mapped to changing body position, self-care, and major life areas; with the level of precision varying across items.

### Mixed methods assessment of ND10 and NDI by patients

Of the 78 patients completing both the NDI and the ND10, a greater number strongly agree that the ND10 was easy to read when compared to the NDI (84% versus 68%; *p* < 0.05). There was strong or moderate agreement that both measures were “easy to read”, 94% for the NDI and 98% for the ND10. Similar numbers strongly agreed that the NDI and ND10 contain relevant content (48% versus 45%); with overall rating of item relevance being higher for the ND10 (90%) versus the NDI (84%) (*p* < 0.05). More people found the ND10 easy to answer in comparison to the NDI: 72% versus 66% strongly agreed (NS), and 96% versus 86% agreed (*p* < 0.05). Some respondents reported that response options did not make sense to them—this was reported by 8% for the NDI and 4% for the ND10. Neither questionnaire was seen as providing undue burden to patients since 82% of respondents reported that the NDI was the right length, and 14% said it was too short. Similarly, 74% reported that the ND10 was the right length and 18% said it was too short.

A substantial number of patients (43% for the NDI and 48% the ND10) reported that these measures did not ask enough about the impact of their neck pain on their life.

### Cognitive interview findings

The specific comments raised by respondents are listed in Supplementary File 2. Several themes arose in these comments. Respondents identified multiple issues that were important to them, but that were not covered on the questionnaires. Many respondents noted that specific impairments, such as movement or strength, were not being assessed. These concerns about the need to consider other constructs reflect that this study focused on evaluating specific functional PROMs—and were not taken as problems with the PROMs themselves.

Similarly, many respondents noted that specific types of pain or sensory disturbance were not assessed by one or both measures. Several respondents noted the importance of numbness/tingling, and that these symptoms were bothersome, but not painful. Another domain that respondents noted as being absent was social function. Things like intimacy, relationships, finances, etc. were relevant impacts that were not addressed by either the NDI or ND10. For the ND10 these would be outside the defined construct of functional disability, but important considerations for quality of life. Interestingly these items do appear on some of the other PROMs in Table 3, but this was deemed problematic in terms of construct clarity and unidimensionality.

Many respondents noted that their disability issues had changed over time, and that this may have affected how they calibrated items. Reducing or replacing recreation or work activities to avoid pain were cited as examples. Some relayed that they experienced deterioration in status after their initial recovery, and these temporal changes made it challenging to answer questions, or to have confidence that PROMs adequately reflect their experience with neck pain. Many respondents noted that the items did not reflect the complexity of their neck problem.

The main specific concern raised about the items that did fit within the construct of disability was about response options. There were multiple respondents who found the response options on the NDI difficult to understand, not descriptive of their status, or to contain conflicting options. Conversely, while this complaint did not occur on the ND10, a few respondents noted that the response options were less defined which made it difficult for them to calibrate. This reflects the different approaches on the two measures. The NDI has detailed response options that are often double-barreled or not mutually exclusive, whereas the ND10 has simple anchors that are used for all items, but there is no clarification of how to define “a little” or “moderate”. This contrast was noted by patients.

### Item distributions and floor/ceiling effects

**Fig. 1:**
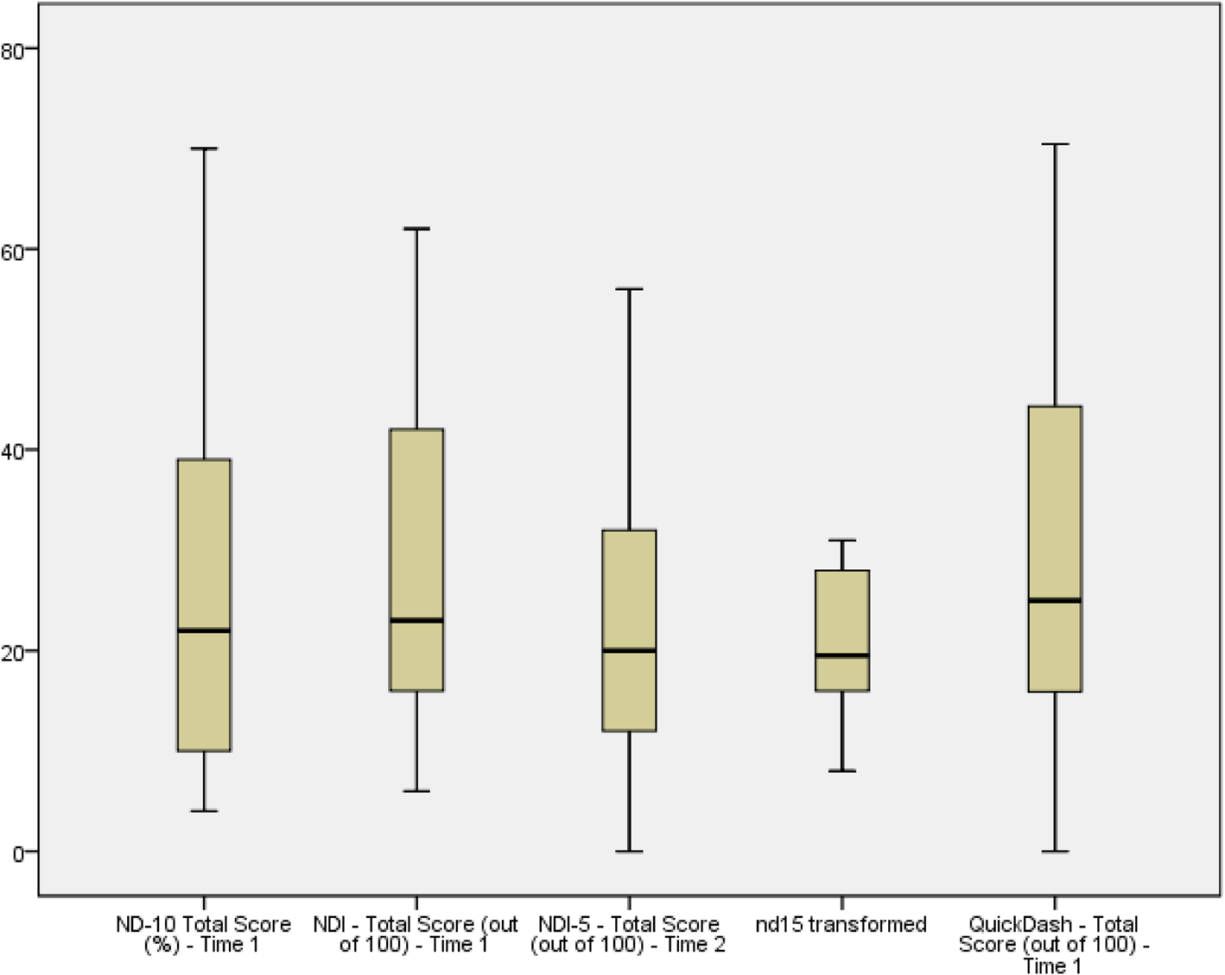
Score Distributions for studied PROMs (/100)

There were no ceiling effects for either the ND10, NDI, QDASH, or NDI-5 as none of the patients scored 90 or higher on any of the measures. There were minor concerns about floor effects as the percentage in the bottom 10% was 8%, 18%, 11%, and 23%, respectively. The NDI and NDI-5 exceeded the floor threshold set at 15%. The box plots reflect a similar mean score estimation across the different instruments, with wide confidence intervals excepting the Rasch-transformed version of the NDI-5 (Fig. 1).

### Reliability/agreement

The internal consistency of the ND10 was 0.93. The ICC for re-test reliability was 0.87 (95% CI 0.76 – 0.93). The Bland and Altman plot indicated minimal bias between test and re-test of the ND10 (0.6 mean difference) with Limits of Agreement (18.6 to—17.4). See Additional file 3: Supplemental Figure A for the Bland and Altman graphs. The SEM was 3.2 and the MDC90 was 7.5.

### Construct validity

Correlations followed constructed hypotheses in that the ND10 was strongly correlated with other measures of neck and arm pain and disability (NDI, NDI-5, DASH) and moderately related to the SANE (Table 4).

**Table 4:** Convergent validity: comparing the ND10 with related measures

Comparative measure	Time 1 (n)	Time 2 (n)	ND10- Time 1	ND10- Time 2
NDI	78	36	0.90^*^	0.86^**^
NDI-5	73	35	0.91**	0.90**
NDI-5 T	70	34	0.87	0.85
QDASH	51	30	0.89^**^	0.87^**^
SANE	51	30	-0.51^**^	-0.36^*^

*NDI* Neck Disability Index, NDI-5 is the raw NDI-5 score and T is the score transformed based on the Rasch model; *QDASH* Quick DASH, *SANE* Single alphanumeric (Neck) evaluation, *n* number of participants completing measure, statistical significance *p < 0.05, ** *p* < 0.01

The constructed hypothesis was supported indicating that the ND10 and the other measures were highly discriminative between patients who rated themselves as more or less than 50% of normal (Table 5).

**Table 5:** Known group validity: comparing patients who self-reported being than 50% normal

Measure	SANE > 50	SANE 0–50	Difference	*p*-value
ND10/100	27.2	42.9	15.7	0.008
NDI/100	27.2	43.1	15.9	0.001
QDASH/100	29.1	49.4	20.3	0.002
NDI-5 T/50	20.0	26.3	6.3	0.003

### Structural validity (factor analysis)

**Fig. 2:**
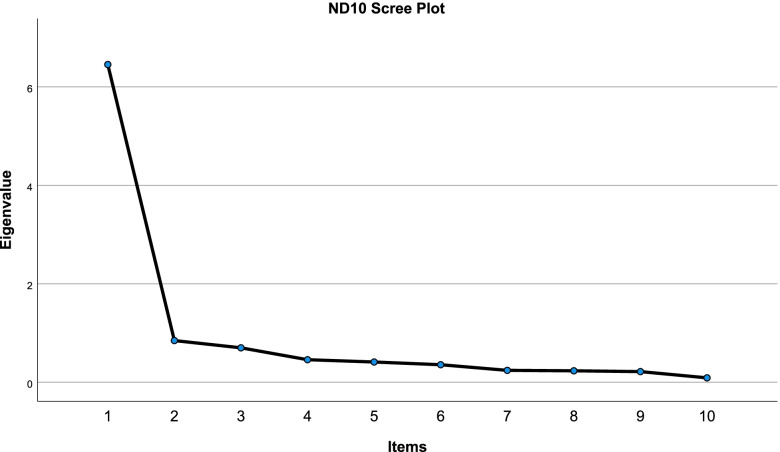
ND10 scree plot

All items loaded on one factor explaining 65% of the variance with a clear demarcation of one factor in the SCREE plot (Fig. 2).

## Discussion

This study provides evidence that the ND10 can provide understandable, relevant, reliable, structurally sound, and discriminative scores representing neck-related disability. While there are multiple PROMs that could be used for people with neck pain, the uniqueness of the ND10 is that it was developed to solely focus on neck-related functional disability, whereas other commonly used measures combine symptoms, pain interference, and other constructs within a single scale. We used iterative quantitative and qualitative work to establish the content validity and usability of the ND10. Content validity analyses was studied using item comparison, classification in ICF and perspective, patient questionnaires and cognitive interviews; and these findings were triangulated during development. The conceptual clarity of the construct being measured, and its constituent items may be the most critical aspect of ND10 development, and is an aspect most sparsely attended to in development of many other neck-related PROMs. COSMIN (COnsensus-based Standards for the selection of health Measurement Instruments) has recently provided more detail highlighting the importance of rigor in content validity [45] and development of item content coding and cognitive interviewing methods provides enhanced methodological support [41].

Although the other PROMs used in the study performed well from a quantitative psychometric view, concerns about content validity of the other neck-related PROMs were apparent in the lack of clear construct definition since items crossed multiple domains and often focused more on pain interference than function. From a health literacy perspective, the readability and relevancy were better than the NDI, and the preference of patients were favourable. Thus, the ND10 may be preferable for clinicians or researchers who wish to distinguish construct of function and pain as recommended by core outcome recommendations [75]. It may also be easier for patients to complete—this is important given the extent to which health literacy is a problem in many clinical contexts. With the move towards identifying core sets of constructs to be measured in musculoskeletal research and practice, the importance of separating pain and disability in separate constructs has become clearer [75–77]. Overall, the ND10’s psychometric properties were better than other neck-related PROMs in terms of establishing a clear conceptual construct and focusing in functional (dis)ability. It was better than the NDI in terms of patient relevance and health literacy and in avoiding floor effects. The ND10 was similar to the NDI and DASH in terms of its convergent association with other measures and ability to discriminate between known groups. Preliminary factor analysis, based on one sample, supported that the ND10 is unidimensional, which has been problematic in other neck-related PROMs including the NDI [33].

An outcome measure which focuses distinctly on disability can be important where it is the focus of a specific treatment or a specific discipline, e.g., rehabilitation. For example, in patients with chronic pain, treatment programs often target improved function without an expectation of substantial improvements in pain [78]. The development of the ND10 was not to diminish the importance of pain as an outcome measure. Conversely, we think that a brief functional neck-specific measure, like the ND10, allows space in patient contact time for a more thorough multi-dimensional pain assessment using a valid pain-specific outcome measure.

Our findings suggest that some of the limitations in previous measures that we hoped to address were successfully mitigated in our new outcome measure. Our prior work indicated the importance of the upper extremity [27, 58] in neck disorders, concerns about high rates of missingness items due to relevancy issues for some items [79], and the importance of considering health literacy during development. Our qualitative interviews indicated the most consistent concern with the NDI was a lack of clarity in the response options. The previous neck PROMs compared at a content level in Table 3 have response options that are longer, have a great cognitive burden, and are sometimes double-barreled or not mutually exclusive. These issues were commonly noted by patients as reasons that it was difficult to calibrate their responses to the NDI in our cognitive interviews. We designed the ND10 to be very simple and brief (118 words on ND10 versus 783 on ND1). Health literacy and cognitive burden are partially related to the number and complexity of words, but also to the format in which information is presented. Therefore, our use of a consistent response options and icons to represent direction were used to improve health literacy. A few of the respondents noted that the ND10 response options being brief meant that they were more open to interpretation. This is inevitable given the choices made for a streamlined format.

Some ND10 items reflect important issues raised by patients in qualitative interviews and surveys [27, 58] that were not included on the NDI, e.g., lifting and carrying a heavy object, putting something on a high shelf, and overhead work. These items create different types of strain on the neck and represent common tasks of daily life. Other issues we encountered during development indicate that items may have a “shelf-life”. For example, the NDI which was developed more than 2 decades ago asks patients about difficulty reading a book. However, many people now primarily read electronic devices. Although the way people read has changed, the ability to read and communicate with text remains an important human function. Therefore, our rewording of a reading item was designed to be more inclusive of different ways that this function is performed. One of the problematic items due to high rates of missingness on the NDI item is the driving item [33, 56]. Driving tends to leave out specific segments of the population, e.g., in some countries women are not allowed to drive; lower income people may not be able to afford vehicles; age restrictions may limit who can drive; and people with comorbidities may have medical reasons for not being allowed to drive. Thus, the driving item inherently represents a form of selection bias. However, the ability to move around in society is an important human function, and many forms of transportation can be difficult for patients with neck disorders. Therefore, this item was included in a more inclusive format by using “drive or ride” and different modes of transportation as exemplars.

Patients did not indicate concerns about the burden of either the NDI or the ND10, and some felt these PROMs were too short. Patients in a qualitative study [27] and our cognitive interviews wanted PROMs to reflect the full scope of the problems they experience. Several patients commented that the measures did not tap into important impacts of their life. Some of these issues were outside of the target construct of functional disability. This indicates the importance of using multiple PROMs to reflect the different constructs important to patients, particularly when these have been defined by core sets [43]. For example, mental symptoms and social/emotional functioning are important but should be measured in separate well-validated PROMs specific to those constructs. Patients in this study may not have understood that typically we would be measuring a larger suite of PROMs within a clinical study or clinical interaction. CATWAD (Core Outcome Domain Set For Whiplash-Associated Disorders) distinguished pain, recovery, and functional disability as separate constructs [43]. Several issues raised by patients reflected recovery or other domains within quality of life. The Satisfaction and Recovery Index [59, 80] is an example of a measure designed to measure recovery following musculoskeletal trauma. Many of the issues that patients raised as missing constructs from the ND10 and NDI fell within the construct measured on The Satisfaction and Recovery Index (e.g., intimacy, life roles). Patient interviews conducted in this study confirm CATWAD findings about the importance of considering both functional disability status and perceived recovery.

We observed that some patients had unique concerns that they felt were important to communicate, but that were not represented on any of the PROMs evaluated. No outcome measure can capture all issues important to every patient. Patients wanted clinicians to understand the complexity of their neck pain. Listening to patients helped us recognize the importance of allowing space to express individual issues qualitatively when responding on an outcome measure. Therefore, we added an open text box to the ND10 where patients can communicate what they want others to know. Although this does not contribute to the score, it is potentially useful in clinical practice since one of the important consequences of implementing outcome measures should be better communication with patients.

The reliability of the ND10 of 0.87 was high, even though the re-test interval was relatively long for some participants (mean 8.5 days; range 4–25) and did not exclude people under treatment. We attribute this to the measure itself and the chronic nature of the patient’s neck disorder. A minimal detectable change of 7.5 points compares favorably with other PROMs. We speculate that test–retest reliability can be influenced by the re-test interval, the acuity of the condition, and the extent to which the construct being measured is stable and definable by patients. We anticipate that future studies that more rigorously assess whether patients have remained stable and use more consistent test–retest intervals might find an even higher reliability coefficient.

The development of a new PROM is justified when there are no PROMs for an important construct or there are serious flaws in existing PROMs. These rationales apply for the ND10 development since previous PROMs lack conceptual clarity, content validity, or failed to adequately incorporate patient perspectives. The ND10 addresses a core construct recommended by an international consensus as being important for patients with neck pain [43]. Despite all of these favourable findings, we recognize it can be difficult to transition to a new PROM. Although there are conceptual flaws with existing neck PROMs, their long-standing use—particularly with respect to the NDI—means that legacy measures have pools of comparative data and familiarity, which may make some people reluctant to change their current usage patterns.

Although this study reports the findings of a multi-stage process, there are limitations in our work. We did not provide the full suite of psychometric evidence. Important future investigations include fit to the Rasch model and responsiveness studies; as well as widespread cross-cultural translation. Although we found excellent reliability and factor structure, the sample sizes were relatively small for these analyses, and future studies in larger samples are needed for greater precision and confidence. A clear understanding of utility of any new PROMs only becomes apparent over time after it has been tested in multiple contexts and populations.

## Conclusions

This study led to the development of a reliable and valid measurement PROM, the ND10, designed specifically for assessing neck-related functional disability. Overall, the findings are supportive of the content validity and suggest strong clinical measurement properties. The ND10 is provided by open access from the developer/copyright owner (J MacDermid: jmacderm@uwo.ca at https://www.lawsonresearch.ca/hulc/outcome-measures) so that it is freely available for use where a simple measure of function is needed for patients with neck pain or disability. It should be used in combination with a pain scale and measures of other salient constructs to reflect multiple aspects of health outcomes and quality of life.

## Supplementary Information

**Additional file 1:Supplementary File 1.** ND10 with scoring instructions.

**Additional file 2:Supplementary File 2.** Record of patient comments about specific questionnaires in cognitive interviews and actions taken.

**Additional file 3:Supplemental Figure A.** Bland-Altman graph demonstrating the mean difference in test and retest scores (0.6) and the limits of agreement (18.6 to -17.4).

## Data Availability

The datasets used and analyzed during the current study are available from the corresponding author upon reasonable request. The data are not publicly available due to ethical and privacy restrictions.

## Abbreviations

CATWAD: Core Outcome Domain Set for Whiplash-Associated Disorders
COSMIN: COnsensus-based Standards for the selection of health Measurement Instruments
DASH: Disabilities of the Arm, Shoulder and Hand
ICC: Intraclass correlation coefficients
ICF: International Classification of Functioning, Disability and Health
IPC: Item Perspective Classification
ND10: Neck Disability 10 (10-item measure of neck-related disability)
NDI: Neck Disability Index
NPR: Numeric Pain Rating Scale
OLBPI: Oswestry Low Back Pain Index
PROMs: Patient-reported outcome measures
QDASH: Quick Disabilities of the Arm, Shoulder and Hand
SANE: Single Assessment Numeric Evaluation
