# Supplementary material for: Development and validation of the ND10 to measure neck-related functional disability
Source: BMC Musculoskelet Disord. 2022 Jun 23;23:605. doi: 10.1186/s12891-022-05556-7 (PMC9219202; doi:10.1186/s12891-022-05556-7)
Supplement: Supplementary file 1 — Additional file 1:Supplementary File 1. ND10 with scoring instructions. [file 12891_2022_5556_MOESM1_ESM.docx]

Supplementary File 1. ND10 with scoring instructions

**Name: __________________________­­­­­­__________________** **Date: ________________________**

Please place a **mark (X)** in the box that ­­describes how much **difficulty** you had **over the past week** for each of the activities listed below **because of your neck**.

|  | **No difficulty**  **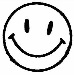** | **A little difficulty** | **Moderate difficulty** | **A lot of difficulty** | **Extreme difficulty** | **Unable to do at all**  **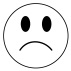** |
| --- | --- | --- | --- | --- | --- | --- |
| **Get washed and dressed** |  |  |  |  |  |  |
| **Lift and carry heavy things** |  |  |  |  |  |  |
| **Read** (book, paper, tablet, computer or phone) |  |  |  |  |  |  |
| **Do my usual work** |  |  |  |  |  |  |
| **Drive or ride** (car, bus, train, bicycle etc.) |  |  |  |  |  |  |
| **Do my usual recreation or sports** |  |  |  |  |  |  |
| **Concentrate on tasks** |  |  |  |  |  |  |
| **Sleep** |  |  |  |  |  |  |
| **Place something on a high shelf** |  |  |  |  |  |  |
| **Do overhead work**  (like change light bulbs, wash walls) |  |  |  |  |  |  |
|  |  |  |  |  |  |  |
| Are there things you want to share about difficulties you have because of your neck problem?  Scoring Instructions:  The Total ND10 Score is out of 100 and can be computed as the sum of the 10 items X 2. The score can be computed as a % of the answered items when item(s) are missing, e.g., when 1 item missing - sum/45 X 100% | | | | | | |

The ND10 MacDermid 2018
