# Supplementary material for: Development and validation of the ND10 to measure neck-related functional disability
Source: BMC Musculoskelet Disord. 2022 Jun 23;23:605. doi: 10.1186/s12891-022-05556-7 (PMC9219202; doi:10.1186/s12891-022-05556-7)
Supplement: Supplementary file 2 — Additional file 2:Supplementary File 2. Record of patient comments about specific questionnaires in cognitive interviews and actions taken. [file 12891_2022_5556_MOESM2_ESM.pdf]

Supplementary File 2. Record of patient comments about specific questionnaires in cognitive interviews and actions taken.

Patient comments		Measure relevance			
		NDI		ND10	
	Interpretation	Raised or Applicable	Gap or Not	Raised or Applicable	Change Made
Did not ask about symptoms and if they are constant or occasional. Did not specifically ask about type of pain and effectiveness of treatment modalities.	Does not address temporal changes in symptoms; or health services	R	G	N*	N*
It didn't ask about any type of weight on my neck. I cannot tolerate even the tiniest amount of weight around my neck. Even the weight of a bulky coat collar is too much. A small camera on a strap or small binoculars cannot be worn on a strap around my neck.	Does not address touch sensitivity	R	G	N*	N*
Neck pain affected my emotions and stress. These were overlooked on the survey.	Does not address emotional or financial status	R	N*	N*	N*
Impact on financial situation not addressed	Financial situation a mediating factor not an outcome	R	N*	N*	N*
I have neck pain on both sides. The right side is aggravated by leaning or sleeping on it, while the right side is aggravated as well, but also by movement.	Does not address differences on between sides; complexity of problem beyond item nuances	R	N	R	N
My biggest issue is turning my head, talking to someone on my left or turning my head when driving and none of this was asked (Does not address impairment; specifics of why driving difficult not specified)	The ND10 and NDI both address driving/transportation but do not specifically state head motion as the limiting factor	X*		X*	
No questions about how the neck problem affects self-esteem or relationship with significant others. Basically, the psychological component is missing. No questions with regards to stigmatization. What about doctor's knowledge of neck injuries and pain management? Of course, since I don't know the purpose or parameters of the study who knows what questions are relevant. (Social function)	Social function out of domain definition of physical function	A	N	X	N
Quality of life - important issues like intimacy, day-to-day living and the effect of the pain. Not just work or movements involved in lifting or	Quality of life out of domain definition, items do not cross or encompass all	X	N	X	N

Patient comments		Measure relevance			
		NDI		ND10	
	Interpretation	Raised or Applicable	Gap or Not	Raised or Applicable	Change Made
looking down (e.g. unloading a dishwasher, hammering, walking, running, working out, gardening, etc.).	possible items but patients may indicate important gaps				
Should ask<br>- if bending, turning or twisting effect your neck,<br>- are you currently taking any prescriptions or<br>- ask about treatment	Aggregating factors and medical care tracking out of scope for an outcome measure.	R	X	X	X
I was young when I injured my neck. Now I am feeling the effects. Comes and goes, but the range of motion is jeopardized every day. (temporal effects of injury- response shift)	Temporal effects- response shift… Not addressed in most cross-sectional outcome assessments- can be addressed using specific response shift techniques	R	N	X	N
I currently have numbness in my hand and tingling in my neck - this is NOT painful, but does affect my ability to do things. I found the survey just asked about pain, technically, the numbness is not painful.	Symptoms in domain for NDI; but numbness/sensory disturbances are not addressed; symptoms not within the domain of the ND10.	R	G	X	X
It did not ask enough about continuing to do activities despite pain. I was mainly think about the work question, if you had pain when you started your job then you are used to working with the pain and your work has likely not actually been impacted by the pain despite experiencing pain.	Important to measure at work difficulty. NDI does assess pain doing some activities and both ask about work, although pain at work outside scope of ND10	R	G	X	N
It wasn't as relevant to people with long standing chronic pain who may have adapted the behaviors to the pain over time. For example, self-care may be adapted compared to the rest of the population, so what people with neck pain are doing may be less than those without and therefore they have no difficulty with it.	does not assess change activity- response shift	A	N	X	N
it did not ask about being able to turn your head at all	Neither designed to directly measure impairments; although impairments affect function	X	X	X	X

Patient comments	Interpretation	Measure relevance			
		NDI		ND10	
		Raised or Applicable	Gap or Not	Raised or Applicable	Change Made
For example, my neck is usually quite painful if I turn my head to the right.	Neither designed to directly measure impairments	X	X	X	X
… Neck resistance	Neither designed to directly measure impairments	X	X	X	X
Does not mention numbness/tingling/burning sensation; quality of pain	NDI measures symptoms but only pain intensity; not in scooe of ND10	X	G	X	N
I feel that the survey doesn't give options that are always relevant to me, or I felt like my answers weren't honest, because I was trying to make them fit to an option. Both surveys don't ask enough - my pain is transient, but also intense, I can occasionally do an action, and then pain will be triggered hours later and last for a week, but then it settles (unable to capture complexity of the problem)	NDI does not ask about pain behavior; ND10 does not address symptoms - by design.	X	G	X	N
There were some categories that did not apply completely to me. For example, I have modified my recreational activities because of my neck pain. Therefore, I was not sure how to answer the question (current or past activities that I can no longer do) (some questions not relevant; does not account for changes made due to problem (response shift)	Response shift is an important issues but PROM measures status and other techniques used to measure response shift	X	N	X	N
I felt the questions about the different categories were more open ended and so generally thought of them in the same way as the first survey. It would be interesting if you have varied the order of the survey for some participants. For example, questions about my usual way (for example lifting) ...this varies depending on the activity. So, there was more ambiguity relative to the first survey. (filling out 2 may have biased responses)	Experimental design may have affected the issues reported	R	X	R	X
Re: work. I can work, but can no longer work as many hours as I used to. Wasn't sure how to address that.	Response options do not fit patients calibration on the NDI; issue is covered on ND10	R	G	R	X

Patient comments		Measure relevance			
		NDI		ND10	
	Interpretation	Raised or Applicable	Gap or Not	Raised or Applicable	Change Made
Difficult to answer in some instances, due to ambiguity of answers. I felt it did not adequately quantify my situation. Ex. I can do many of the activities listed, but they cause me a lot of pain afterward, didn't feel this survey addressed that aspect. (did not like focus on function without considering pain during activity)	The intended use of the ND 10 would be with a validated pain scale, but this is not clear in the experimental design	X	X	X	X
Headaches - missing option of infrequent SEVERE headaches. (response options did not fit)	NDI response categories have gaps in appropriate responses	R	G	X	X
Difficulty understanding which option, overlap.	Response options not clear	X	G	X	X
Last 2 items did not relate to me (not all items relevant)	Items may not be relevant to all patients	X	N	X	X
I read the options repeatedly to ensure I was understanding the option, it's easy to mark off the wrong answer because it says what you THINK you want to reply. Options aren't always a) most pain, e) least pain which also makes it tricky - but then again, perhaps that is intentional? (response options not clear)	The longer wording of items responses made it more challenging for some participants to be clear about the options	X	G	X	X
Some ambiguity in this survey (ND10), I interpreted the questions/answers in several different ways.	Lack of specifics means that patients have to develop internal calibration	X	X	X	G
It was difficult to distinguish the differences between the choices. (response options not clear) NDI	Similar concern about lack of clarity in response options	X	G	X	X
The questions give multiple answers in one question and are too specific. For example, the combined question: Section 3: Lifting. Pain prevents me from lifting heavy weights, but I can manage light to medium weights if they are conveniently positioned. This can be broken down to be better understood and more precise.	Lack of distinct or comprehensive items on NDI	X	G	X	X
It is too general (ND10). What is the purpose of the survey? To determine whether a person has been affected by the pain or whether s/he is totally disabled? Pain can negatively affect every aspect of a person's life and wellbeing. Pain has a different level of	ND10 not intended to measure pain	X	X	X	X

Patient comments		Measure relevance			
		NDI		ND10	
	Interpretation	Raised or Applicable	Gap or Not	Raised or Applicable	Change Made
severity at different times of the year and even day. Questions are not specific about the time. (not comprehensive; does not consider change over time)					
I like the scale and the easy comparisons (ND10)	Simplicity	X	X	R	R

R – denotes that a respondent made this comment and denoted this in related to a specific PROM (NDI or ND10) or the comment equally applied. G means it is a gap area for that measure. X means that this is not a problem, or not relevant as out of scope.
