# Supplementary material for: Development and validation of the ND10 to measure neck-related functional disability
Source: BMC Musculoskelet Disord. 2022 Jun 23;23:605. doi: 10.1186/s12891-022-05556-7 (PMC9219202; doi:10.1186/s12891-022-05556-7)
Supplement: Supplementary file 3 — Additional file 3:Supplemental Figure A. Bland-Altman graph demonstrating the mean difference in test and retest scores (0.6) and the limits of agreement (18.6 to -17.4). [file 12891_2022_5556_MOESM3_ESM.pdf]

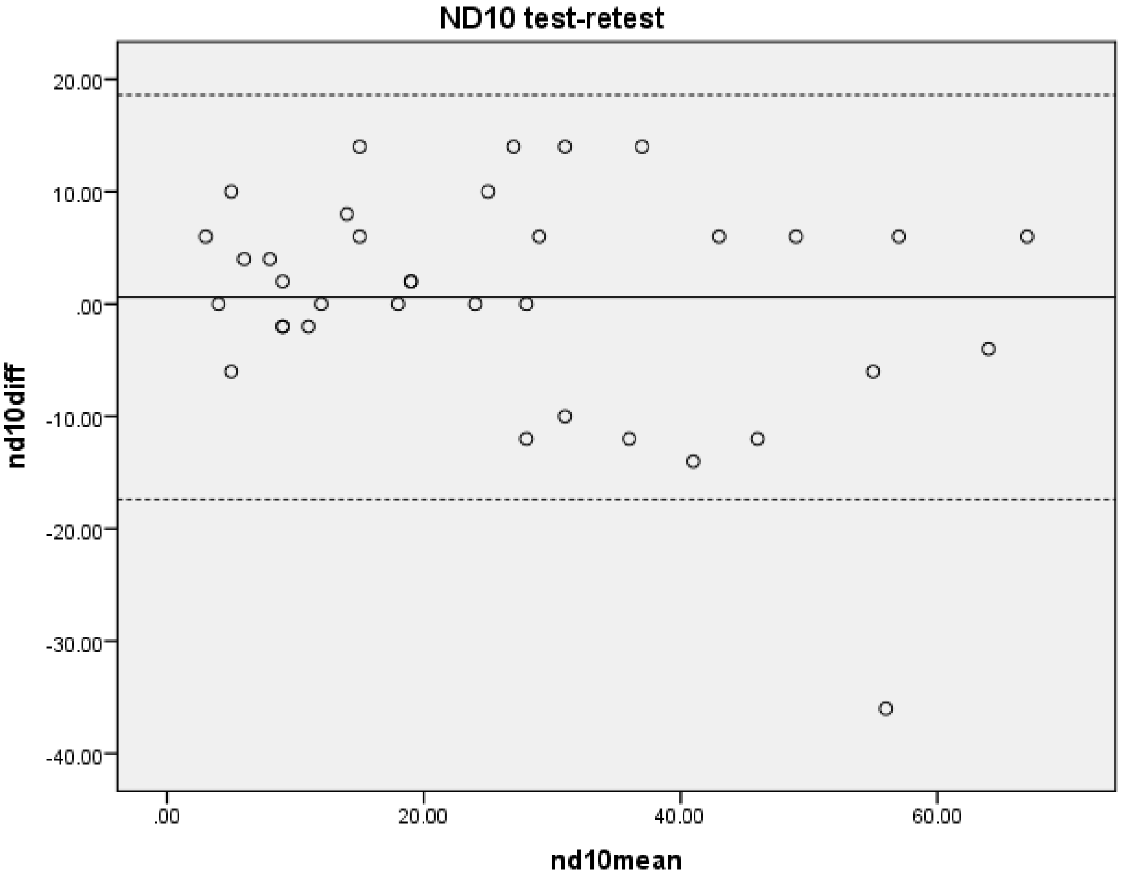

Supplemental Figure A. Bland-Altman graph demonstrating the mean difference in test and retest scores (0.6) and the limits of agreement (18.6 to -17.4)
